# Confining energy migration in upconversion nanoparticles towards deep ultraviolet lasing

**DOI:** 10.1038/ncomms10304

**Published:** 2016-01-07

**Authors:** Xian Chen, Limin Jin, Wei Kong, Tianying Sun, Wenfei Zhang, Xinhong Liu, Jun Fan, Siu Fung Yu, Feng Wang

**Affiliations:** 1 Department of Physics and Materials Science, City University of Hong Kong, 83 Tat Chee Avenue, Hong Kong SAR, China; 2 Department of Applied Physics, The Hong Kong Polytechnic University, Hung Hom, Hong Kong SAR, China; 3 Department of Electronic Engineering, City University of Hong Kong, 83 Tat Chee Avenue, Hong Kong SAR, China; 4 City University of Hong Kong Shenzhen Research Institute, Shenzhen 518057, China

## Abstract

Manipulating particle size is a powerful means of creating unprecedented optical properties in metals and semiconductors. Here we report an insulator system composed of NaYbF_4_:Tm in which size effect can be harnessed to enhance multiphoton upconversion. Our mechanistic investigations suggest that the phenomenon stems from spatial confinement of energy migration in nanosized structures. We show that confining energy migration constitutes a general and versatile strategy to manipulating multiphoton upconversion, demonstrating an efficient five-photon upconversion emission of Tm^3+^ in a stoichiometric Yb lattice without suffering from concentration quenching. The high emission intensity is unambiguously substantiated by realizing room-temperature lasing emission at around 311 nm after 980-nm pumping, recording an optical gain two orders of magnitude larger than that of a conventional Yb/Tm-based system operating at 650 nm. Our findings thus highlight the viability of realizing diode-pumped lasing in deep ultraviolet regime for various practical applications.

## Abstract (web summary)

A general approach to maximize upconversion luminescence in stoichiometric lanthanide lattices is lacking. Here, Chen *et al.* report a NaYbF_4_:Tm lattice and demonstrate fine-tuning of energy migration by controlling dimensions of the crystal lattice, highlighting their potential for deep ultraviolet lasing.

## Graphical abstract

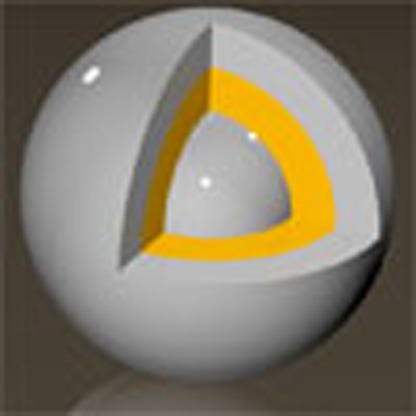

The construction of functional materials with designable optical properties is fundamentally important for scientific research and technological applications in diverse fields encompassing energy, environment and biomedicine^1^,^2^,^3^,^4^,^5^,^6^,^7^,^8^,^9^,^10^,^11^. Given the constraints in designing materials using different combinations of elements, nanoscale manipulation of matters has become a promising alternative to the creation of novel functional materials^12^,^13^,^14^,^15^,^16^,^17^,^18^,^19^. Particularly, by taking the advantage of size confinement effects, the energy band structure in semiconductors can be precisely modified to offer size-tunable emission wavelengths^20^,^21^. Despite the attractions, the size effect is largely unexplored in lanthanide-doped upconversion nanoparticles, which represents an important family of optical materials characterized by large anti-Stokes shift, narrow emission bandwidths and long excited-state lifetimes.

Photon absorption and emission in upconversion nanoparticles are due to the lanthanide dopants localized on the lattice sites^22^,^23^. In principle, a high concentration of lanthanide dopants enhances upconversion processes as a result of an elevated capacity to sustain excitation energy^24^,^25^,^26^,^27^. However, a high lanthanide content also enhances energy migration through the crystal lattice, which usually leads to a depletion of the excitation energy^24^,^28^. To minimize nonradiative energy losses, energy migration is typically inhibited by doping low concentrations of lanthanide ions^27^,^28^ or by using special host lattices^24^. Currently, there lacks a general approach to maximize upconversion luminescence in stoichiometric lanthanide lattices.

In this work, we describe an investigation of energy migration in a nanosized NaYbF_4_ lattice. We demonstrate fine tuning of energy migration through controlling the dimensions of the crystal lattice. Our mechanistic investigation reveals a spatial confinement of energy migration that prevents energy loss to the crystal lattice and increases the local density of excitation energy. Through the use of Tm^3+^ ion as an energy accumulator, the excitation energy can be maximally amassed to generate intense ultraviolet emissions on near-infrared excitation. We show that the technological advancement may revolutionize the fabrication of cost effective and compact diode-pumped solid-state deep ultraviolet lasers that are useful for environmental, life science and industrial applications^29^.

## Results

### Synthesis and characterization

**Figure 1:**
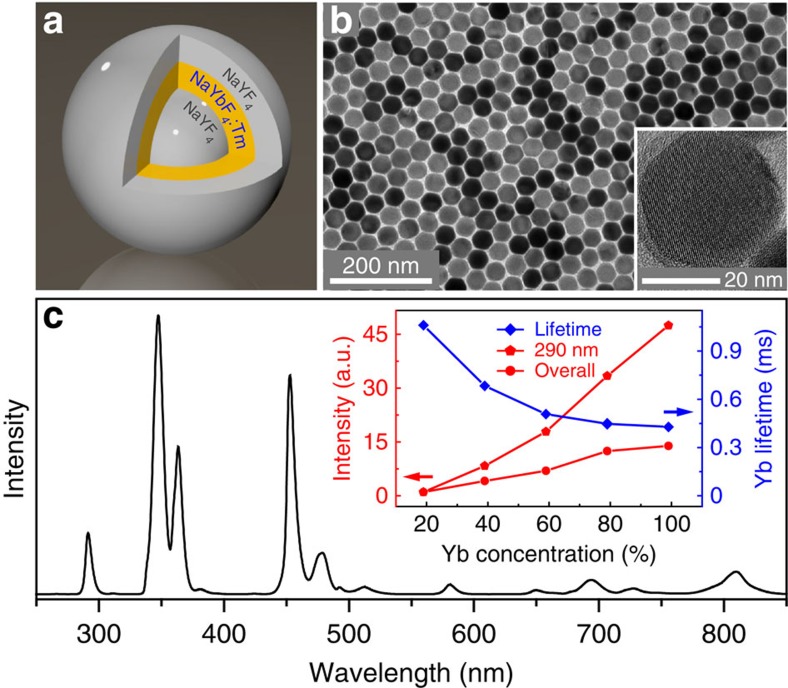
Deep ultraviolet upconversion in core–shell–shell nanoparticles. (**a**) Schematic design of a NaYF_4_@NaYbF_4_:Tm@NaYF_4_ core–shell–shell nanoparticle for confining the migration of excitation energy generated in the Yb^3+^ ions. (**b**) TEM image of the as-synthesized nanoparticles. Inset: high-resolution TEM image reveals single-crystalline nature of the particle. (**c**) Upconversion emission spectrum of the nanoparticles under 980 nm excitation (CW laser diode, 20 W cm^−2^). Inset: ^2^F_5/2_ lifetime of Yb^3+^, emission intensity at 290 nm and integrated emission intensity over 250–850 nm range versus dopant concentration of Yb^3+^, respectively. Note that the solid lines are intended to guide the eye.

As a proof-of-concept experiment, we confined Yb^3+^ ions in the inner shell layer of a hexagonal phase NaYF_4_@NaYbF_4_:Tm@NaYF_4_ host (Fig. 1a), which is known to render high upconversion efficiencies^3^. In our study, the concentration of Tm^3+^ was fixed at 1 mol% to maximize upconversion emission in the ultraviolet region (Supplementary Fig. 1). We did not employ a NaYbF_4_:Tm@NaYF_4_ core–shell structure because existing synthetic protocols give essentially no access to sub-10 nm β-NaYbF_4_ nanoparticles of a tunable particle size, which is critical prerequisite for assessing the effect of confining energy migration on upconversion. Although Yb-doped β-NaYF_4_ nanoparticles with small feature size can be synthesized by several complimentary methods^23^,^30^, pure β-NaYbF_4_ tends to form big particles (Supplementary Fig. 2a) due to rapid growth of the crystal^31^. Through the use of preformed NaYF_4_ core nanoparticle as a template, the growth of the NaYbF_4_ crystal can be effectively regulated, thereby offering exquisite control over the lattice dimensions (Supplementary Note 1). Note that it is also critical to enclose the Yb sublattice in an inert protection layer (that is, NaYF_4_) because a NaYF_4_@NaYbF_4_:Tm core–shell structure yields luminescence that is substantially weak due to surface quenching (Supplementary Fig. 2b,c).

The nanoparticles were fabricated by a layer-by-layer epitaxial growth process (Supplementary Note 1). Transmission electron microscope (TEM) images (Fig. 1b) reveal a highly uniform morphology of the nanoparticles with an average size of 38 nm. High-resolution TEM (inset of Fig. 1b) and X-ray powder diffraction (Supplementary Fig. 3) experiments confirm the single-crystalline nature of the as-synthesized nanoparticles with a hexagonal phase. To verify the formation of the multilayer structure, we intentionally doped Gd^3+^ ions in the inner shell layer to create a contrast under electron energy loss spectroscopy analysis. The difference in the elemental distribution of Y and Gd clearly indicates the presence of multiple core–shell interfaces (Supplementary Fig. 4).

Figure 1c shows a representative upconversion emission spectrum of NaYF_4_@NaYbF_4_:Tm@NaYF_4_ nanoparticles on 980 nm excitation with a continuous wave (CW) laser diode at a power density of 20 W cm^−2^. The spectrum consists of characteristic emission peaks that can be assigned to ^1^I_6_→^3^H_6_ and ^3^F_4_ (290 and 350 nm), ^1^D_2_→^3^H_6_ and ^3^F_4_ (360 and 450 nm), ^1^G_4_→^3^H_6_ and ^3^F_4_ (475 and 650 nm) and ^3^H_4_→^3^H_6_ (800 nm) transitions of Tm^3+^, respectively. Both the violet and overall emissions surpass that of the NaYF_4_@NaYbF_4_:Tm/Y@NaYF_4_ counterparts comprising lower Yb^3+^ contents (inset of Fig. 1c and Supplementary Figs 5 and 6). Notably, Tm^3+^ emission at 290 nm originating from a five-photon upconversion declined by over 45-fold when the Yb^3+^ concentration dropped to 19 mol%, which in conventional systems typically produces the maximum emission of Tm^3+^ ions^24^,^28^.

### Confinement of energy migration

We attribute the observations to confined migration of excitation energy within the nanoshells, which prevents the excitation energy from travelling a long distance at a high Yb^3+^ concentration (99 mol%). The absence of long-distance energy migration is likely to suppress energy loss to the crystal lattice accounting for luminescence quenching. Furthermore, the localization of excitation energy raises the rate of energy transfer to a nearby Tm^3+^ activator, which facilitates the multiphoton upconversion process.

**Figure 2:**
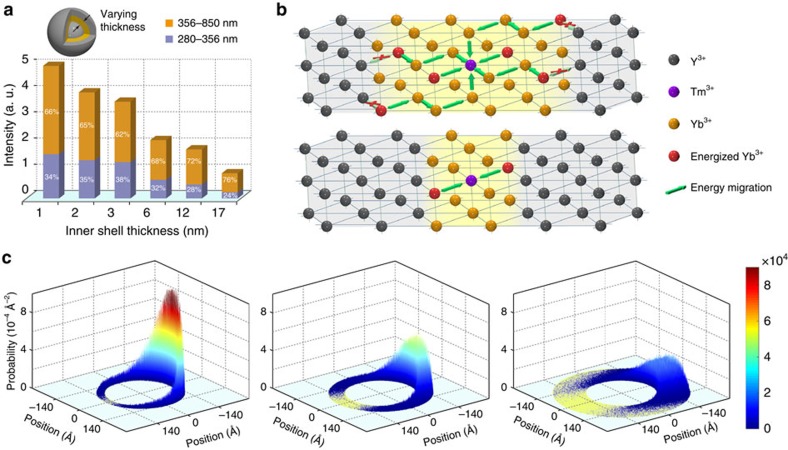
The effect of inner shell thickness on upconversion. (**a**) Upconversion emission intensity versus inner shell thickness (1–17 nm). The emission intensities were calculated by integrating the spectral intensity of the emission spectra that are normalized to the absorption of Yb^3+^ at 980 nm. (**b**) Schematic illustration showing proposed energy transfer from Yb^3+^ to Tm^3+^ in Yb-sublattice of varying dimensions. Note that only partial lattice sites are shown for clarification. (**c**) The probability of finding the excitation energy on the equatorial section of core–shell–shell nanoparticles of varying inner shell thickness. With increasing inner shell thickness (from left to right panels), the energy migrates to a larger area and the probability of finding the excitation energy in the vicinity of the starting point drops significantly.

An assessment of a series of NaYF_4_@NaYbF_4_:Tm@NaYF_4_ nanoparticles of varying inner shell thickness from 1 to 17 nm verified the spatial confinement of energy migration (Supplementary Fig. 7). Luminescence decay studies reveal a markedly lengthened lifetime of the Yb^3+^ by a factor of over nine with decreasing inner shell thickness from 17 to 1 nm (Supplementary Fig. 8a), confirming the suppression of energy loss to the host lattice in thin shells. In contrast, the decay time of localized Tm^3+^ transition was only increased by less than twofold for the same series of samples (Supplementary Fig. 8b), suggesting that the defect density in the host lattice were marginally modified. Therefore, the suppressed depletion of excitation energy of Yb^3+^ may be dominantly ascribed to the spatial confinement of energy migration, which reduces the quantity of defects accessible to the Yb sublattice. In line with the reduced energy loss, we observed a steady enhancement of upconversion emission, especially the part in the 280−356 nm range that originates from the five-photon process, accompanied by a decrease of the inner shell thickness (Fig. 2a; Supplementary Fig. 9). The slight drop in the ratio of five-photon upconversion to overall emission for substantially thin shells (that is, 1 and 2 nm) can be attributed to the reduced amount of Yb^3+^ ions in the vicinity of a Tm^3+^ activator (Fig. 2b), which limits the quantity of energy that can be captured by a Tm^3+^ activator in a photo cycle. It is noted that the spatial confinement of energy migration also plays a role in cubic phase NaYbF_4_:Tm (1%)@NaYF_4_ core–shell nanoparticles (Supplementary Fig. 10)^32^, implying that the geometry of the host lattice is not a formative factor in restraining the migration of excitation energy.

To shed more light on energy migration in the core–shell–shell nanostructure, we calculated the probability distribution function of excitation energy as a function of space within the Yb shell. For simplification, we assumed that the excitation energy randomly hops in the inner shell layer through the Dexter energy transfer (Supplementary Note 2). As shown in Fig. 2c, the energy migrates to smaller areas with decreasing thickness of the Yb shell from 12 to 6 and 3 nm, supporting reduced coupling of excitation energy to defects. The high probability of finding the excitation energy in a thin Yb shell further validates a favourable energy transfer to an adjacent Tm^3+^ activator.

### Lasing through upconversion

**Figure 3:**
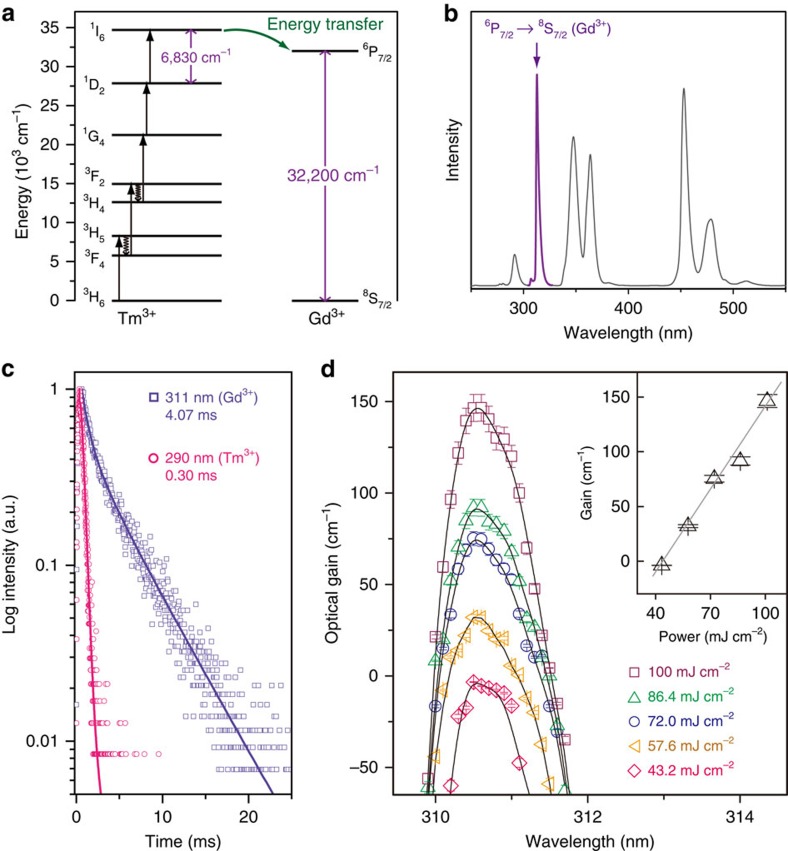
The effect of Gd^3+^ doping on the deep ultraviolet upconversion. (**a**) Simplified energy level diagram showing the energy gaps in Tm^3+^ and Gd^3+^ activators, respectively. (**b**) Upconversion emission spectra of the core–shell–shell nanoparticles co-doped Gd^3+^ (30 mol%) in the inner shell layer (CW laser diode, 20 W cm^−2^). (**c**) A comparison of the excited state lifetime between ^1^I_6_ state of Tm^3+^ and ^6^P_7/2_ state of Gd^3+^ in the NaYF_4_@NaYbF_4_:Tm/Gd (1/30%)@NaYF_4_ core–shell–shell nanoparticles. (**d**) Gain spectra of the nanoparticles in **b** as a function of excitation power (pulse laser). The inset gives the corresponding optical gain versus pump power at a wavelength of 310.5 nm. The straight line is the linear regression of the measured data. Error bars shown represent the s.d.'s from five sets of repeated measurements.

To facilitate the use of the upconversion nanoparticles as gain media for lasing applications, we further developed a Gd^3+^ doping method for optimizing the optical properties (Supplementary Fig. 11). We used a 3-nm inner shell for the study due to a high-intensity ratio of five-photon upconversion emission and a relatively low mass ratio of the optically inert NaYF_4_ layers. Gd^3+^ ions are able to extract the excitation energy of Tm^3+^ ions and generate a new emission peak centred at around 311 nm, owing to the reasonably matched energy levels (that is, ^6^P_7/2_ level of Gd^3+^ and ^1^I_6_ level of Tm^3+^) (Fig. 3a,b). Importantly, the large energy gap (32,200 cm^−1^) in Gd^3+^ favours the preservation of the excitation energy as supported by time decay studies (Fig. 3c). The long-lived excited state contributes to high optical gains of around 150 cm^−1^ through five-photon upconversion (Fig. 3d), which is comparable to that of the GaN-based semiconductor quantum wells operating in deep ultraviolet at room temperature^33^,^34^. The optical gain is also two orders of magnitude higher than that of a conventional Yb/Tm-based system operating at 650 nm through three-photon upconversion^35^. Moreover, efficient emission is attained at a high Gd^3+^ concentration (30 mol%; Supplementary Fig. 12), which provides abundant carriers to sustain optical gains at high excitation powers without saturation (Fig. 3d).

**Figure 4:**
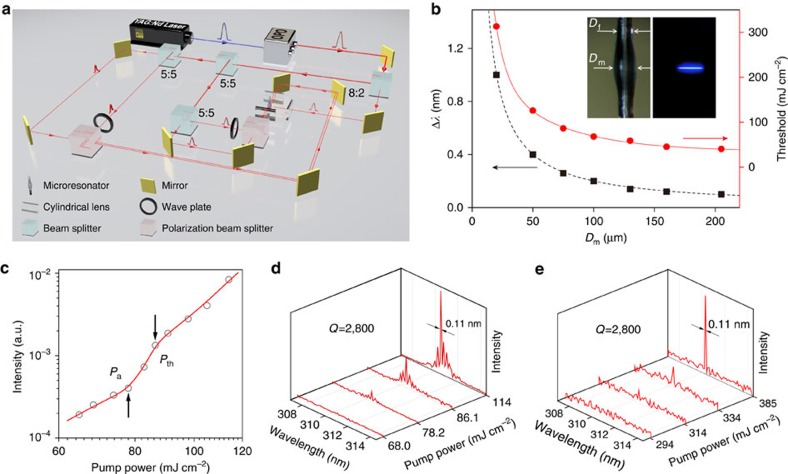
Upconversion lasing characteristics of the microresonator. (**a**) Schematic diagram of the optical set-up for the five-pulse excitation scheme. (**b**) Plots of measured Δ*λ* and *P*_th_ of the microresonator as a function of *D*_m_. The red and black lines are fitted and calculated curves, respectively. The insets show photographs of the microresonator with and without optical excitation. *D*_f_ and *D*_m_ denote the diameters of the fibre and microcavity, respectively. (**c**) Logarithmic plot of output intensity versus excitation power of a microresonator with *D*_m_=75 μm. The red line is fitted curve. (**d**) The corresponding lasing spectra at different excitation power (*D*_m_=75 μm). (**e**) Single mode lasing spectra measured from a microresonator with *D*_m_=20 μm.

To realize lasing emission, we constructed a five-pulse pumping scheme to excite the upconversion process (Fig. 4a). The pulse excitation scheme is primarily intended to alleviate the problems of catastrophic optical damage and the thermal effects associated with CW excitation, which terminates upconversion lasing actions. Furthermore, the five-pulse system is advantageous over the single-pulse system for pumping the multiphoton upconversion (Supplementary Fig. 13), as a result of improved alignment with the excitation process where the absorption of photons occurs sequentially^36^.

The laser cavity was fabricated by coating a drop of silica resin containing the nanoparticles onto a standard optical fibre. Driven by surface tension, the silica resin tends to form a bottle-like microresonator, which supports whispering gallery modes at a thin equatorial ring near the surface of the microresonator (Fig. 4b). Notably, the emission features such as mode spacing and threshold pump power of the microresonator can be readily tuned by controlling the diameter (*D*_m_) of the resonator (Fig. 4b)^37^,^38^, which provides a general platform for assessing the optical characteristics of the upconversion nanoparticles.

We validated the lasing action by measuring the optical emission in a typical microresonator (*D*_m_=75 μm) under excitation of varying powers at room temperature. The light input–output curve shown in Fig. 4c exhibits a well-defined nonlinear excitation power-dependent behaviour with three distinct regions separated by two threshold pump powers (that is, *P*_a_=∼78 mJ cm^−2^ and *P*_th_=∼86 mJ cm^−2^). This *S*-like spectrum clearly indicates a transition from a spontaneous emission to an amplified spontaneous emission and to a lasing emission. Figure 4d shows the corresponding emission spectra under various pumping powers. At low pump power (<*P*_a_), a relatively broad spontaneous band is observed. As the excitation power increases slightly above *P*_a_, a sharp peak ascends from the emission spectrum. Through further increases in the excitation power above *P*_th_, well-defined sharp peaks with a linewidth <0.11 nm emerge from the spectrum. The measured mode spacing (0.25−0.27 nm) is in good agreement with the theoretical value (∼0.26 nm), confirming that lasing emissions have been achieved. Notably, single-mode lasing was also obtained by using a thinner microresonator (*D*_m_=20 μm; Fig. 4e). The single-mode emission is a result of a relatively large mode spacing (∼1.0 nm) with respect to the full width at half maximum of the resonant frequency. This narrow gain bandwidth is a unique signature of the upconversion nanoparticles and unlikely to be realized from semiconductor nanostructures. The *Q* factor, which is defined as the ratio of the resonant frequency to its full width at half maximum, was estimated to be ∼2,800, revealing the high quality of the upconversion-based laser system. It is also worth noting that the lasing emission can be readily extended to violet and blue spectral regions with the same upconversion nanoparticles (Supplementary Fig. 14).

## Discussion

Our investigation of energy migration in nanostructured hosts highlights an innovative strategy to manipulating optical transitions in lanthanide-doped upconversion nanoparticles. In addition, it initiates a novel tactic to obtain effective upconversion laser materials in deep ultraviolet regime with very narrow optical gain bandwidth to support single-mode excitation. Emission, in principle, can be tuned to shorter wavelengths (that is, well below 300 nm) by further refining the upconversion process. Hence, our study will lead to the development of near-infrared diode-pumped deep ultraviolet lasers which can avoid the difficulty of shifting the operating wavelength of GaN-based laser diodes below 300 nm (ref. 39), evade using nonlinear optical crystal that requires tight control in optical alignment, antireflective coating and environmental control and adopt inexpensive *Q*-switched near-infrared diode as the pumping source to construct compact, deep ultraviolet lasers for unexplored applications in the fields of information technology, biomedicine and biophotonics.

## Methods

### Nanoparticle synthesis

We synthesized the core–shell–shell nanoparticles using the method described in 16. Additional experimental details are provided in the Supplementary Note 1.

### Optical gain measurement

Net optical gain of the nanoparticle colloid was measured using variable stripe length method^40^. The longer side of a quartz cuvette filled with nanoparticle colloid was excited by a pump stripe with width and length of ∼30 μm and *L*, respectively in the orientation perpendicular to the length of the cuvette. Photoluminescence intensity emitted from the shorter side of the cuvette, *I*_tot_(*λ*), was recorded by the monochromator set-up. The net optical gain, *G*(*λ*), was deduced by fitting *I*_tot_ (*L*, *λ*)=*I*_sp_(*λ*) [exp(*G*(*λ*)*L*)−1]/*G*(*λ*) with the measured values of *I*_tot_(*λ*), where *λ* is the wavelength and *I*_sp_(*λ*) is the spontaneous emission intensity.

### Five-pulse excitation scheme

The optical set-up consists of a Powerlite DLS 9010 *Q*-switched Nd:YAG laser and a continuum Panther EX optical parametric oscillator. A 980-nm laser pulse (6 ns, 10 Hz) with a diameter of ∼10 mm was generated from the optical parametric oscillator system under the excitation of the Nd:YAG laser. By splitting the 980-nm pulse into five equal-power pulses through the use of four beam splitters (that is, one 80/20 and three 50/50 beam splitters), we can obtain a five-pulse (time delay between adjacent pulses is 10 ns) laser beam. This is possible because the pulses are forced to travel in five different distances to obtain a time delay of 10 ns between the adjacent pulses. These five pulses are then combined through two polarization-dependent beam splitters and two *λ*/2 waveplates to form three laser beams. All the laser beams, which are spatially overlapped, are focussed onto a sample through three cylindrical lenses to form a pump stripe of width equal to ∼30 μm. Photoluminescence emission from the sample was collected and analysed by an optical fibre (core diameter of 400 μm) coupled to an Oriel MS257 monochromator attached with a photomultiplier tube. The spectral resolution of the monchromator is about 0.1 nm.

### Fabrication and excitation of microresonators

For the fabrication of the bottle-like microresonator, a bared standard optical fibre was coated with a tiny drop of nanoparticles and silica resin mixture. The prolate surface-tention-induced microresonator was then solidified in an arid and clean atmosphere. The surrounding temperature of the sample was kept at 23 °C to avoid deformation due to the influence of thermal effects. Whispering gallery modes can be excited by optically pumping the equatorial zone of the microresonator. Notably, the pump stripe is oriented perpendicular to the length of the fibre. Light emitted from the surface of the microresonator can be collected through an optical fibre.

## Additional information

**How to cite this article:** Chen, X. *et al.* Confining energy migration in upconversion nanoparticles towards deep ultraviolet lasing. *Nat. Commun.* 7:10304 doi: 10.1038/ncomms10304 (2016).

## Supplementary Material

Supplementary Information Supplementary Figures 1-14 and Supplementary Notes 1-2
