# Supplementary material for: Confining energy migration in upconversion nanoparticles towards deep ultraviolet lasing
Source: Nat Commun. 2016 Jan 7;7:10304. doi: 10.1038/ncomms10304 (PMC4729831; doi:10.1038/ncomms10304)
Supplement: Supplementary Information — Supplementary Figures 1-14 and Supplementary Notes 1-2 [file ncomms10304-s1.pdf]

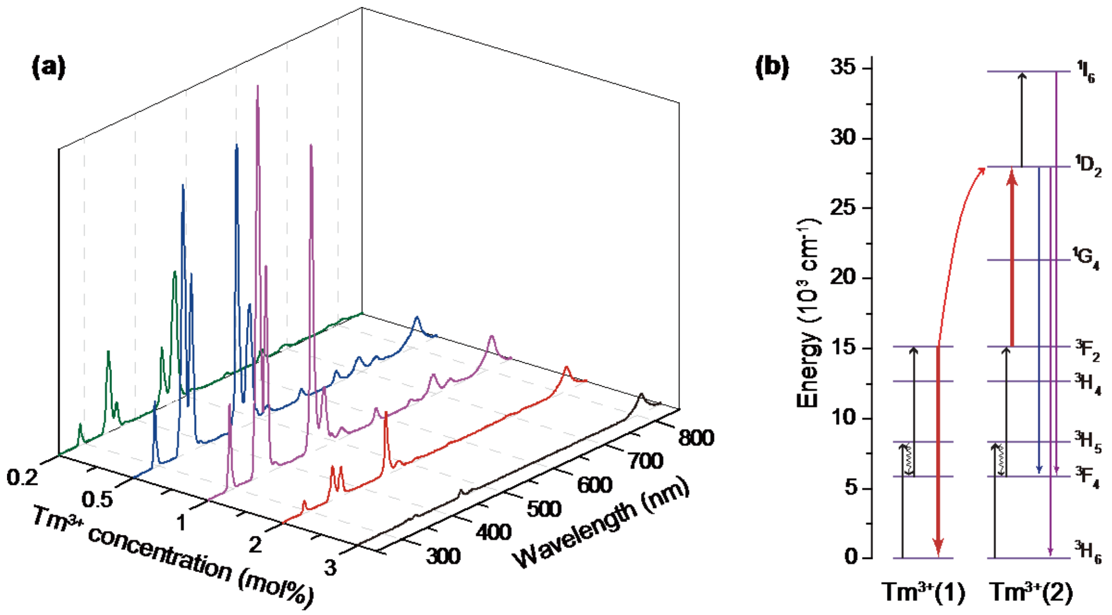

Supplementary Figure 1: (a) Upconversion emission spectra of the $NaYF_4$@$NaYbF_4$:Tm(x%) @$NaYF_4$ core–shell–shell nanoparticles as a function of Tm^{3+} content in the inner shell layer. The spectra were recorded on water dispersions of corresponding nanoparticles (0.03 M) by excitation with a 980-nm CW laser diode at a power density of 20 W cm^{-2}. A relative higher Tm^{3+} concentration (1 mol%) is typically needed to maximize emission in the UV spectral region, probably due to an energy transfer between Tm^{3+} ions that facilitates the population in the $^1D_2$ excited state as proposed in (b). At substantially higher Tm^{3+} concentrations (e.g.; 2 and 3 mol%), the upconversion emission intensity drops significantly due to self-quenching.

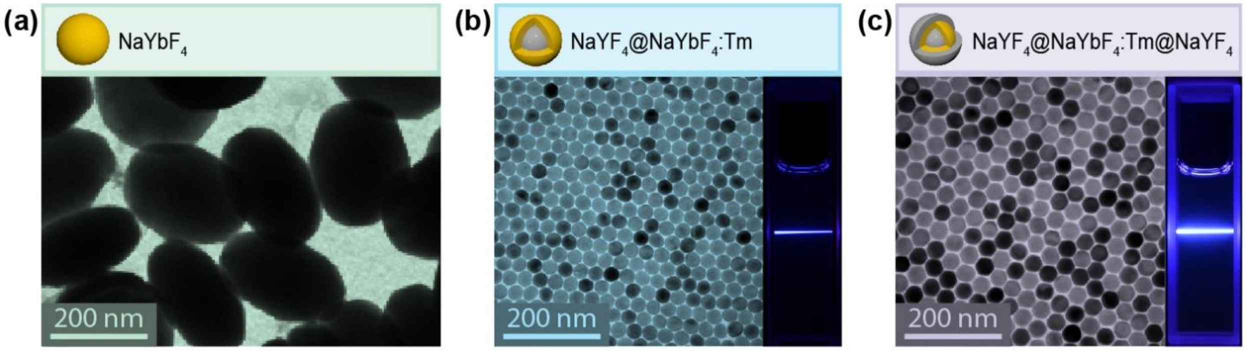

Supplementary Figure 2: Control experiments justifying the need for core–shell–shell structure to confine the excitation energy. (a) $NaYbF_4$:Tm design leads to substantially large particles. (b) $NaYF_4$@$NaYbF_4$:Tm design can lead to small particles size. But the emission is rather weak due to energy dissipation at the nanoparticle surfaces. (c) $NaYF_4$@$NaYbF_4$:Tm@$NaYF_4$ structure render high emission intensity due to elimination of surface quenching effects. Luminescence photos were obtained in cyclohexane solutions comprising 0.02 mol/L Yb^{3+} under 980-nm excitation with a CW laser diode at a power density of 20 W cm^{-2}.

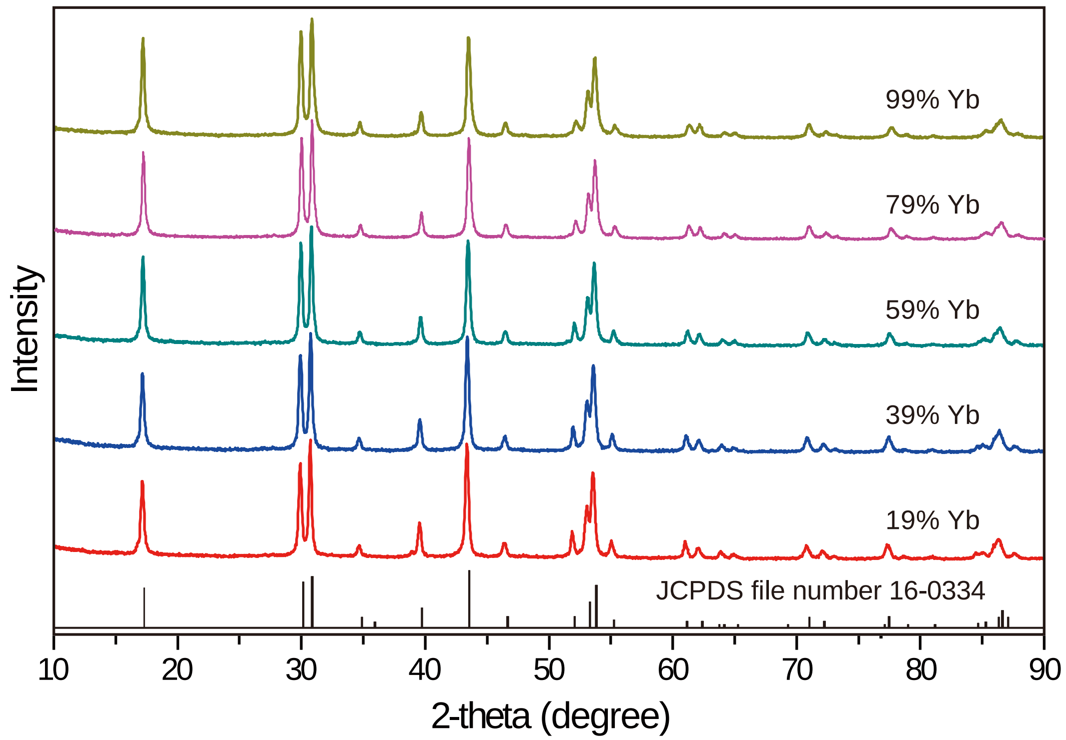

Supplementary Figure 3: XRD patterns of the $NaYF_4$@$NaYbF_4$:Tm/Y(1/x%)@$NaYF_4$ core–shell–shell nanoparticles comprising varying concentration of Yb^{3+} in the inner shell layer. The line spectrum of hexagonal phase $NaYF_4$ crystal (JCPDS standard file number 16-0334) is also included as a reference. The results show pure hexagonal phase of the products.

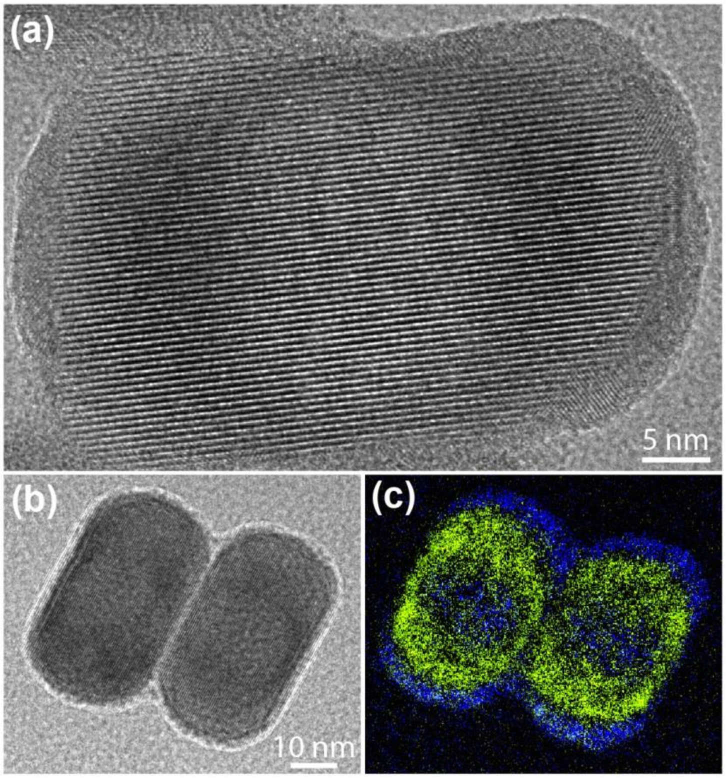

Supplementary Figure 4: (a) High-resolution TEM image of a $NaYF_4$@$NaYbF_4$:Tm/Gd (1/30 mol%)@$NaYF_4$ core–shell–shell nanoparticle reveals single-crystalline nature of the nanoparticle. (b) TEM image of randomly selected nanoparticles for compositional analysis. (c) Element maps of Gd and Y in the nanoparticles shown in (b). Gd^{3+} ions were intentionally doped in the inner shell layer to create a contrast under energy loss spectroscopy (EELS) analysis because Yb and Y are indistinguishable under our experimental setup. The difference in the elemental distributions for Y (blue) and Gd (green) confirms the formation of the core-shell-shell structure. The elongation in nanoparticle morphology is attributed to a large variation in surface energies of different crystal facets caused by the Gd^{3+} dopants.

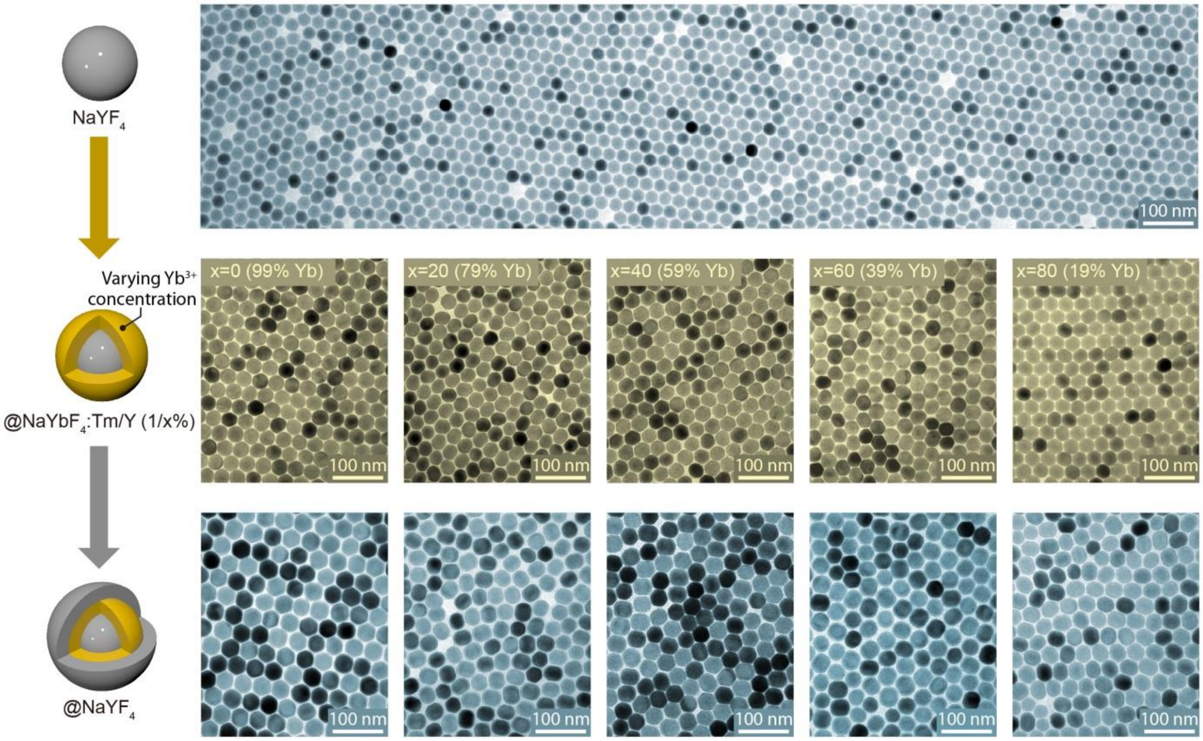

Supplementary Figure 5: TEM images of the $NaYF_4$@$NaYbF_4$:Tm/Y(1/x%)@$NaYF_4$ core–shell–shell nanoparticles comprising varying concentration of Yb^{3+} in the inner shell layer. The core–shell–shell nanoparticle synthesis involves the growth of core particles (~26 nm) through oleate route followed by successive deposition of two epitaxial shells of $NaYbF_4$:Tm/Y (1/0-80 mol%) and $NaYF_4$.

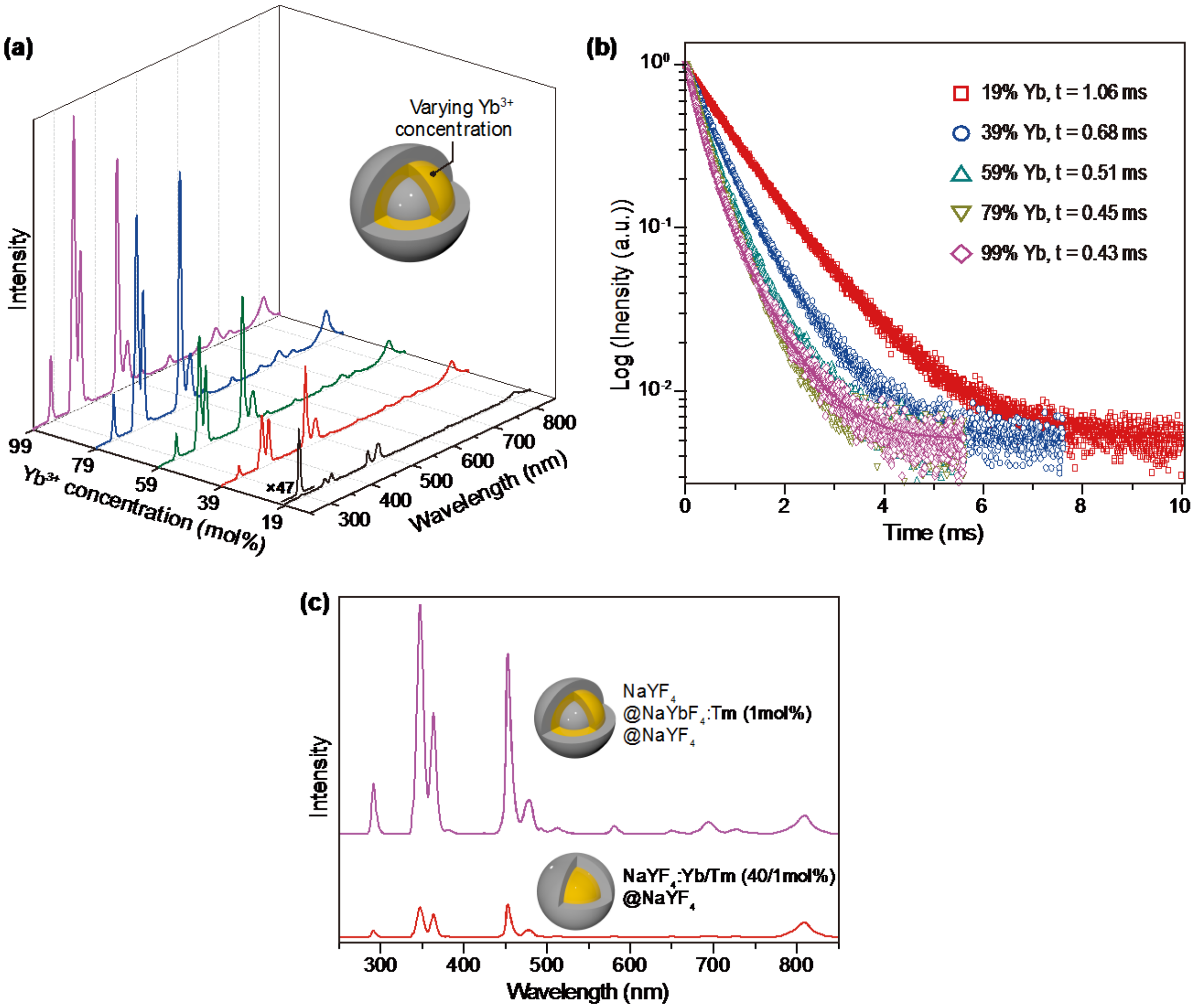

Supplementary Figure 6: (a) Upconversion emission spectra of the $NaYF_4$@$NaYbF_4$:Tm/Y(1/x%)@$NaYF_4$ core–shell–shell nanoparticles as a function of Yb^{3+} content in the inner shell layer. The spectra were recorded on water dispersions of corresponding nanoparticles (0.03 M) by excitation with a 980-nm CW laser diode at a power density of 20 W cm^{-2}. (b) Time decay curves of Yb^{3+} in the core–shell–shell nanoparticles obtained by monitoring the downshifting emission at 980 nm after excitation with a pulse laser at 940 nm. (c) Emission intensity comparison of the $NaYF_4$@$NaYbF_4$:Tm(1%)@$NaYF_4$ and $NaYF_4$:Yb/Tm(40/1%)@$NaYF_4$ nanoparticles. The spectra were recorded under excitation of a 980-nm CW diode laser at a power density of 20 W cm^{-2}. The intensities were normalized to the mass of the emission layer.

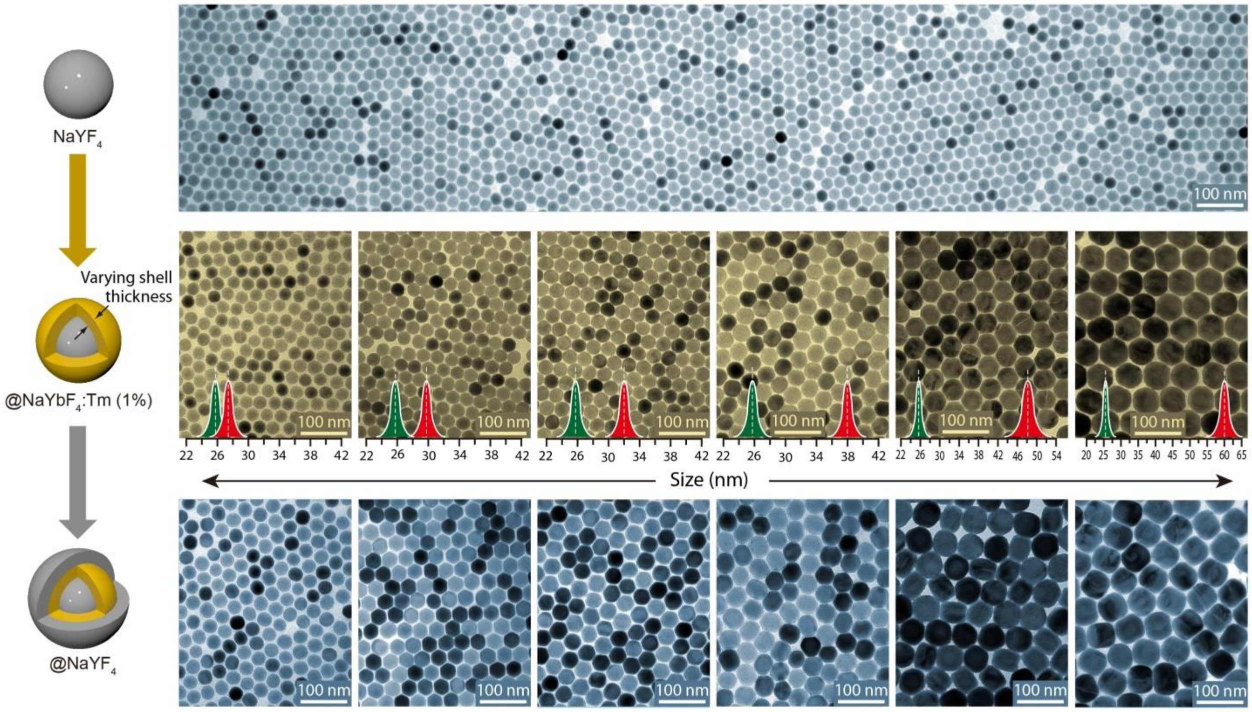

Supplementary Figure 7: TEM images of the $NaYF_4$@$NaYbF_4$:Tm(1%)@$NaYF_4$ core–shell–shell nanoparticles with varying inner shell thickness. Core particles with a diameter ~26 nm were synthesized and separated for following epitaxial shell growth. Inner shell thickness was tuned by adding different amount of shell precursor. The particle size was represented by counting more than 100 particles.

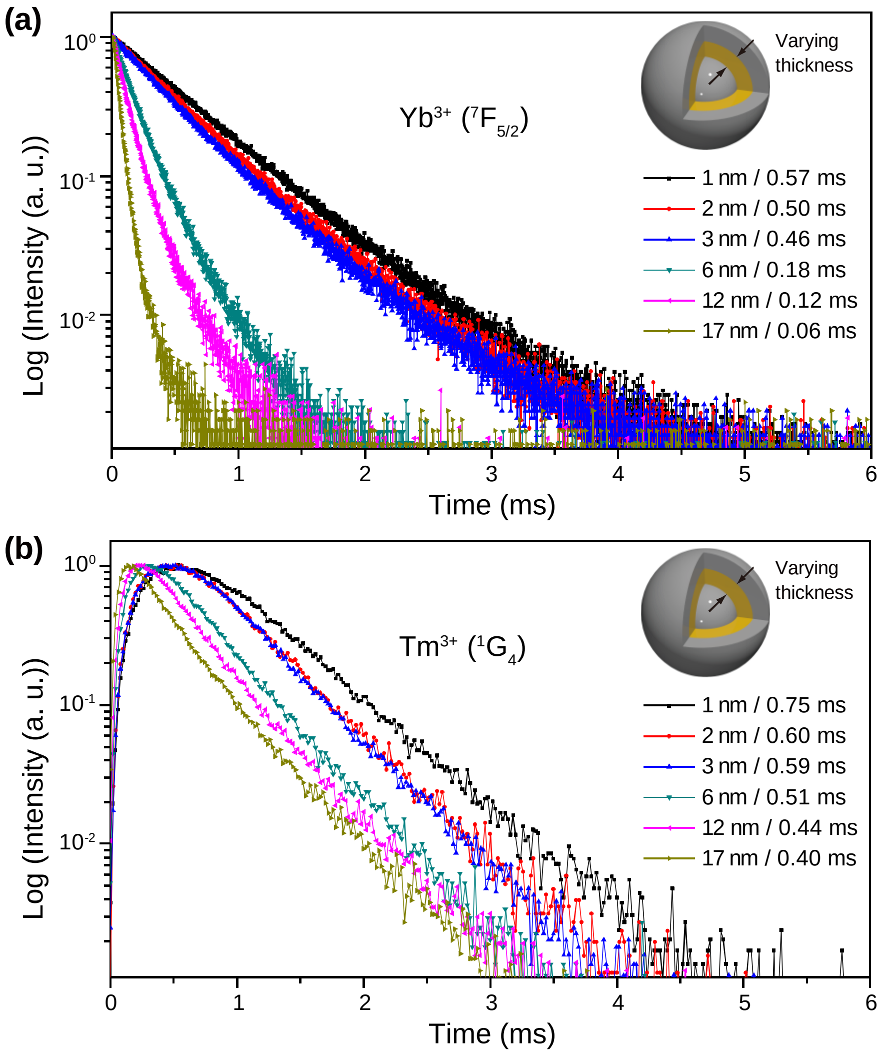

Supplementary Figure 8: A comparison of luminescence lifetimes of (a) $^7F_{5/2}$ state of Yb^{3+} and (b) $^1G_4$ state of Tm^{3+} in the $NaYF_4$@$NaYbF_4$:Tm(1%)@$NaYF_4$ core–shell–shell nanoparticles as a function of the inner shell thickness. The decay curves of localized Tm^{3+} transition ($^1G_4 \rightarrow {}^3H_6$) show marginally dependence on the shell thickness, implying a similar defect density in these nanoparticles. The marked increase in Yb^{3+} lifetime with decreasing inner shell thickness is thus attributed to the spatial confinement of energy migration that suppresses energy loss to the host lattice.

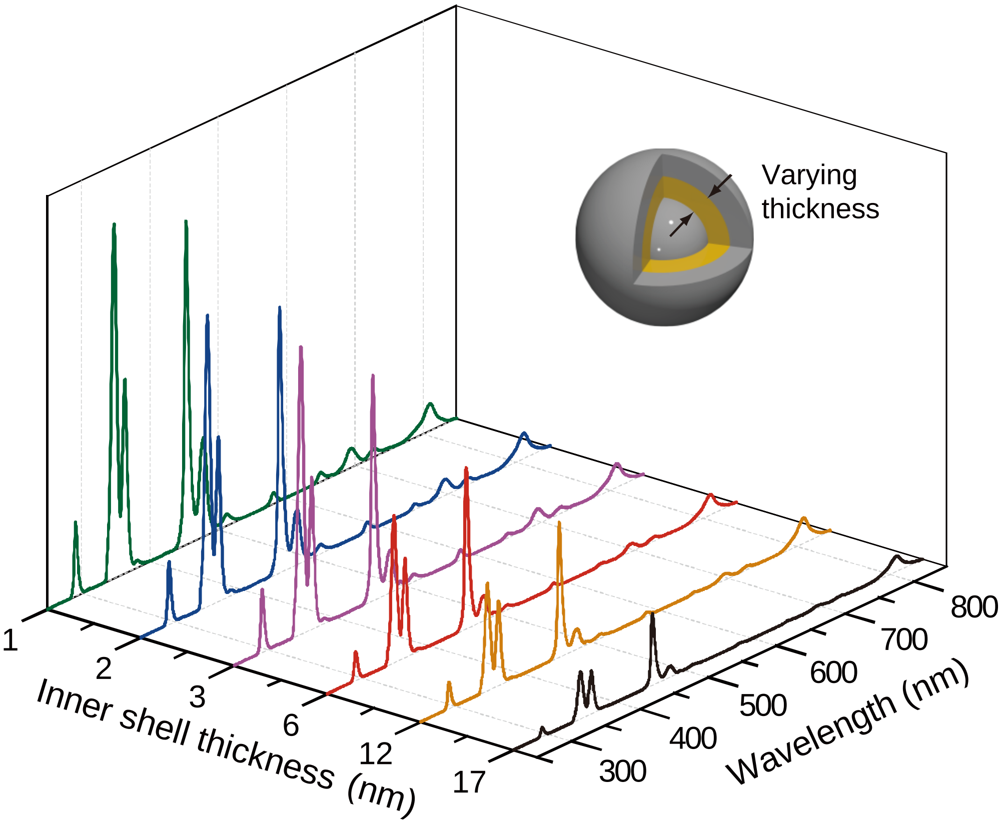

Supplementary Figure 9: Upconversion emission spectra of the $NaYF_4$@$NaYbF_4$:Tm (1%) @$NaYF_4$ core–shell–shell nanoparticles as a function of inner shell thickness under 980-nm excitation (CW laser diode, 20 W cm^{-2}). The spectra were normalized to Yb^{3+} absorption at 980 nm.

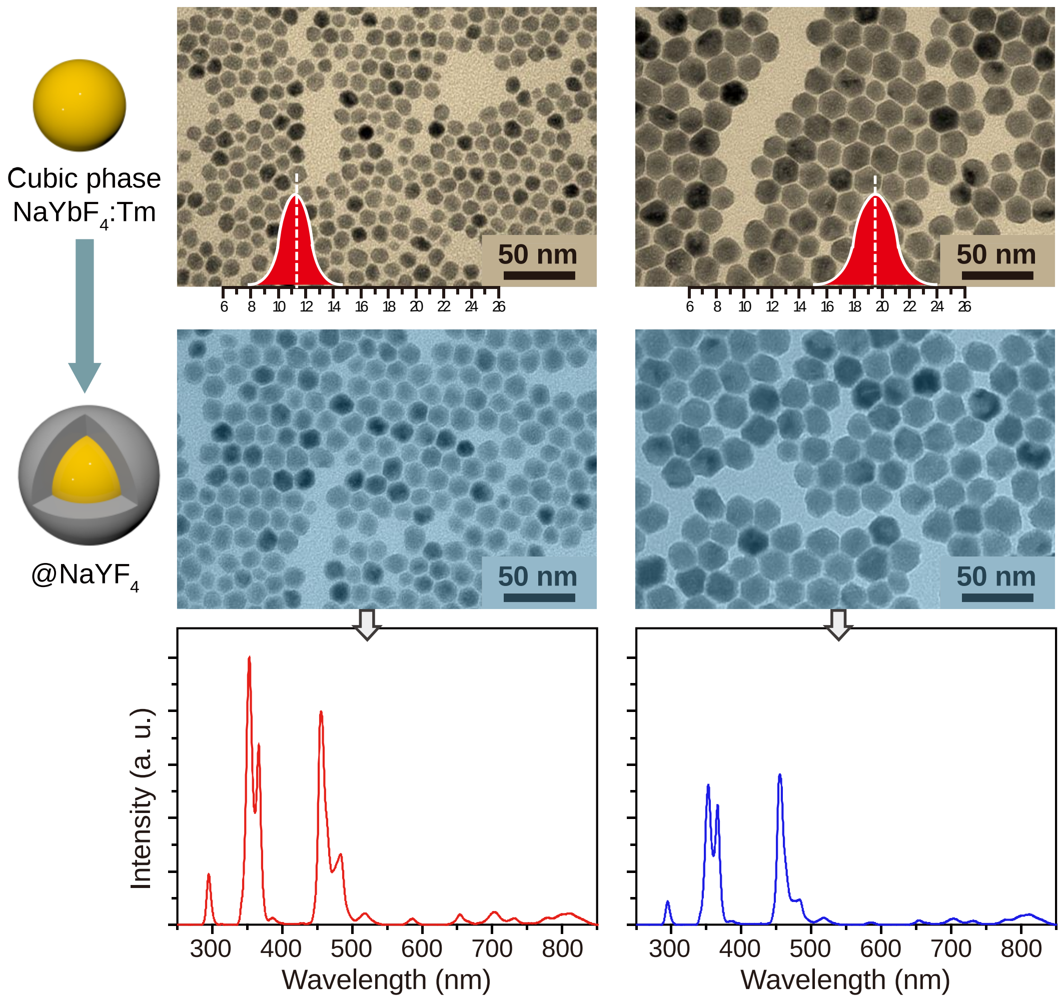

Supplementary Figure 10: Control experiments showing the confinement effect on energy migration in cubic phase $NaYbF_4$:Tm (1%)@$NaYF_4$ core–shell nanoparticles. The emission spectra were normalized to Yb^{3+} absorption at 980 nm. Both the total emission intensity and the ratio of 5-photon emission to total emission are higher in the smaller particles.

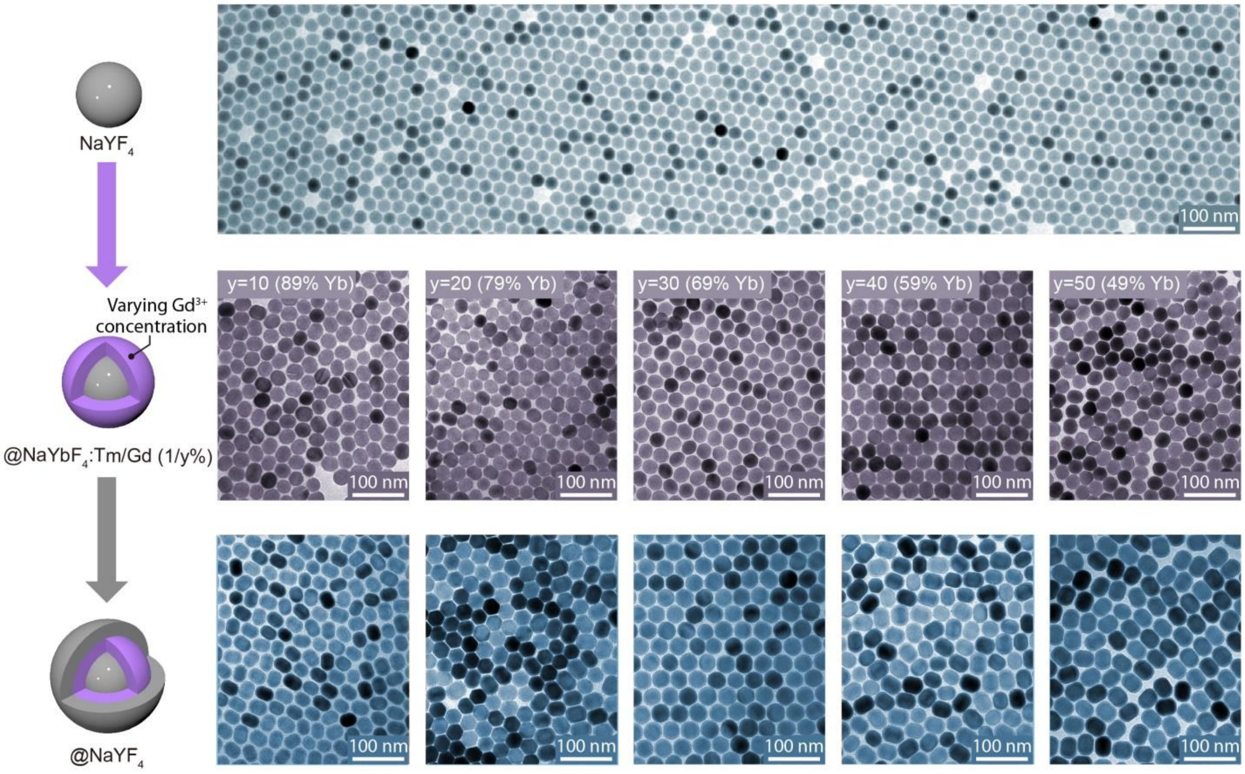

Supplementary Figure 11: TEM images of the $NaYF_4$@$NaYbF_4$:Tm/Gd(1/y%)@$NaYF_4$ core–shell–shell nanoparticles comprising varying concentration of Gd^{3+} in the inner shell layer. The core-shell-shell nanoparticles synthesis involves the growth of core particles (~26 nm) through oleate route followed by successive deposition of two epitaxial shells of $NaYbF_4$:Tm/Gd (1/y mol%) and $NaYF_4$

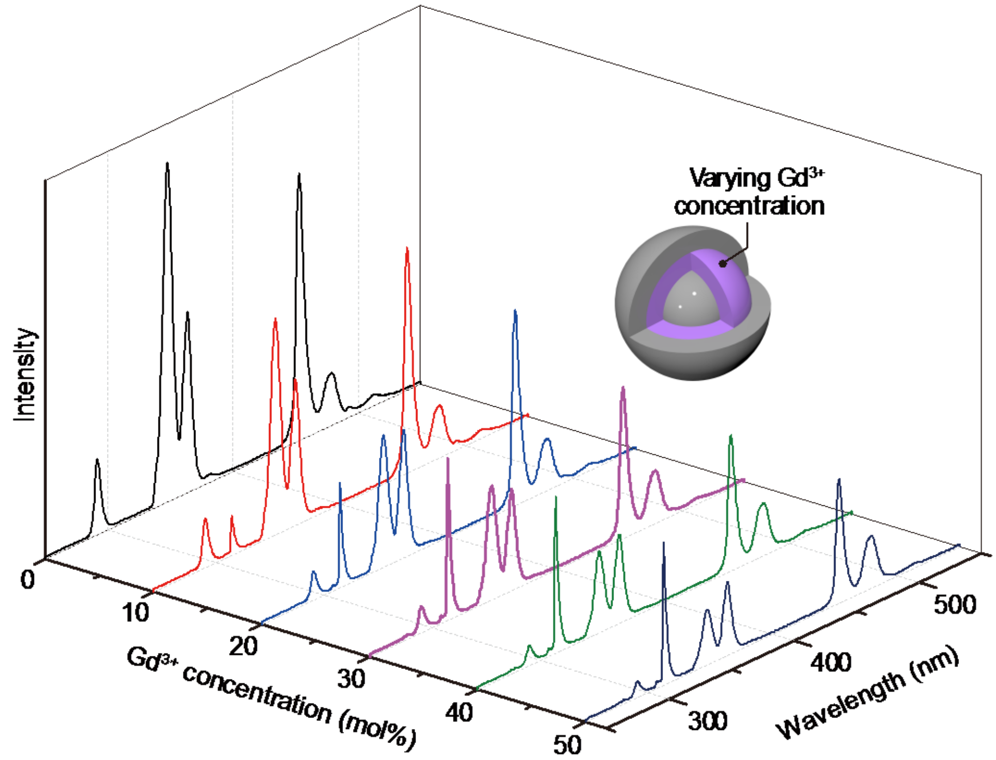

Supplementary Figure 12: Upconversion emission spectra of the $NaYF_4$@$NaYbF_4$: Tm/Gd(1/y%)@$NaYF_4$ core–shell–shell nanoparticles as a function of Gd^{3+} content in the inner shell layer. Emission intensity at 311 nm increased with increasing dopant concentration of Gd^{3+} from 0 to 30 mol%. When dopant concentration of Gd^{3+} was further increased, the emission intensity at 311 nm declined due to the drop in overall emission intensity ascribed to low Yb^{3+} concentrations. The spectra were recorded on water dispersions of corresponding nanoparticles (0.03 M) by excitation with a 980-nm CW laser diode at a power density of 20 W cm^{-2}.

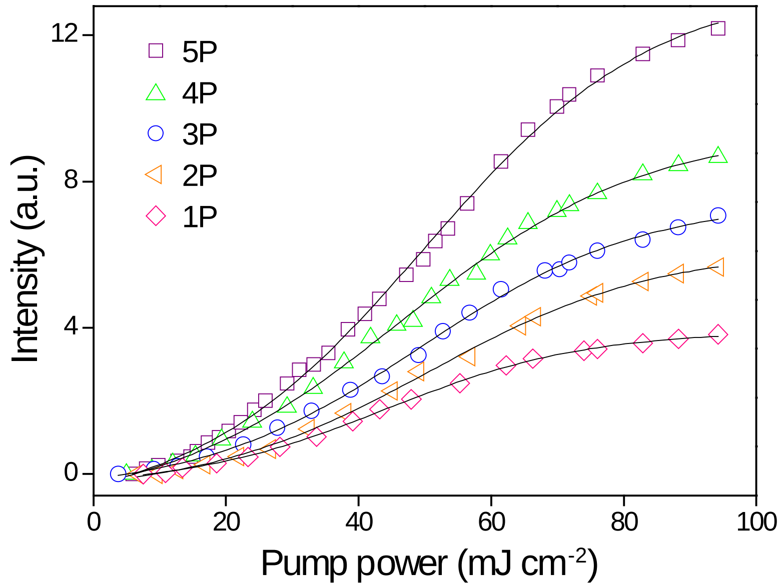

Supplementary Figure 13: Upconversion emission intensity at 311 nm of the $NaYF_4$@ $NaYbF_4$:Tm/Gd (1/30 mol%)$NaYF_4$ core–shell–shell nanoparticles as a function of excitation power for different excitation schemes (i.e.; 1- to 5-pulse trains). We generate a train of pulses to excite the upconversion nanoparticles because we want to 1) obtain high peak excitation power, 2) avoid thermal and catastrophic optical damage, and 3) maximize excitation efficiency especially for the 5-photon upconversion process. The results show that 5-pulse excitation can significantly improve the upconversion emission intensity of the nanoparticles. We therefore used the 5-pulse train excitation scheme throughout the laser experiment.

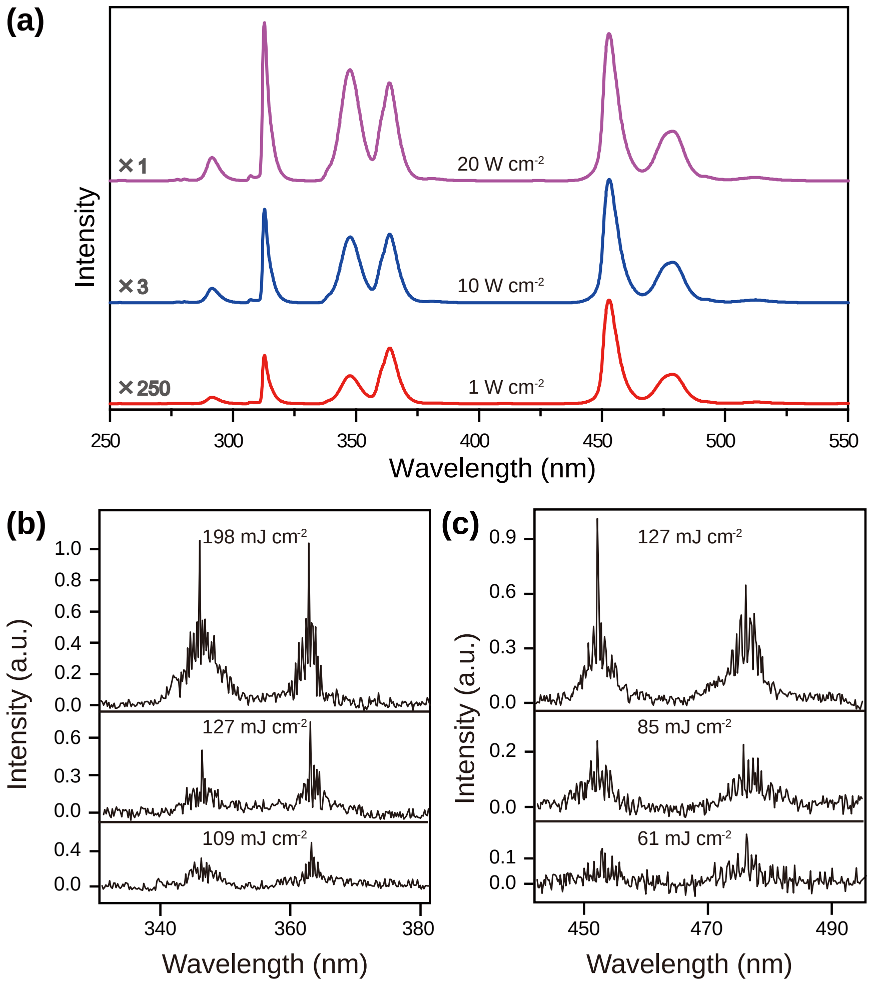

Supplementary Figure 14: The effect of excitation power on emission profiles of the nanoparticles. (a) Plots of the emission spectra versus excitation power density for the $NaYF_4$@$NaYbF_4$:Tm/Gd(1/30 mol%)@$NaYF_4$ core–shell–shell nanoparticles. A low excitation power results in relatively stronger blue emission bands with respect to the ultraviolet ones. (b, c) The corresponding lasing spectra versus excitation power for a cylindrical cavity with D_m = 65 μm in the wavelength ranges of 340–380 nm and 450–490 nm, respectively.

Supplementary Note 1: Nanoparticle synthesis and characterization

Reagents: Ytterbium(III) acetate hydrate (99.9%), yttrium(III) acetate hydrate (99.9%), gadolinium(III) acetate hydrate (99.9%), thulium(III) acetate hydrate (99.9%), sodium hydroxide (NaOH, >98%), ammonium fluoride (NH_4F, >98%), hydrochloric acid (ACS reagent ,37%), 1-octadecene (ODE, 90%), oleic acid (OA, 90%), oleylamine (OM, 90%) were all purchased from Sigma-Aldrich and used as received.

General procedure for the synthesis of the core nanoparticles: Typically, 24-mL water solution of $Y(CH_3CO_2)_3$ (0.2 M) was added to a 250-mL flask containing 36 mL of oleic acid and 84 ml of 1-octadecene. The mixture was heated to 160 ^oC for 120 min to form the lanthanide-oleate precursor solution and then cooled down to 50 ^oC naturally. Thereafter, a mixture of NaOH (12 mmol) and NH_4F (18.96 mmol) in methanol was added and stirred for 120 min. The resultant solution was heated at 100 ^oC for 30 min under vacuum to remove the methanol. After purging with argon, the solution was heated to 300 ^oC and kept for 2 h before cooling down to room temperature. The as-prepared nanoparticles were precipitated by additional of ethanol, collected by centrifugation at 6000 rpm for 3 min, and washed with ethanol for several times. The core nanoparticles are stored in cyclohexane (48 ml) prior to being used for shell coating.

General procedure for the synthesis of core–shell nanoparticles: The shell precursor was first prepared by mixing 2-mL water solution of corresponding lanthanide acetates (0.2 M) with 3 mL of oleic acid and 7 mL of 1-octadecene in a 50-mL flask followed by heating at 160 ^oC for 40 min. After cooling down to 50 ^oC, 5-mL methanol solution of NH_4F (1.58 mmol) and NaOH (1 mmol) was added and stirred at 50 ^oC for 30 min. The resultant solution was heated at 100 ^oC for 30 min under vacuum to remove the methanol. To grow the shell layer, the preformed core nanoparticles (0.6 mmol) were heated with 3 mL of OA and 7 mL of ODE to 300 ^oC under argon, at which time the shell precursor was injected and the temperature was kept for 1 h. The resulting nanoparticles were precipitation by addition of ethanol, collected by centrifugation at 6000 rpm for 3 min, washed with ethanol, and re-dispersed in 6 mL of cyclohexane. Core–shell nanoparticles with different shell thickness can be prepared by controlling the amount of injected shell precursor.

General procedure for the synthesis of the core–shell–shell nanoparticles: The procedure is identical to the synthesis of core–shell nanoparticles, except that the as-synthesized core–shell nanoparticles were used as seeds to mediate the shell growth.

General procedure for the synthesis of cubic phase $NaYbF_4$:Tm@$NaYF_4$ nanoparticles: The procedure is similar to the synthesis of the hexagonal phase counterparts, except that metal trifluoroacetates were used as precursors to thermally decompose in a ternary solvent mixture of OA, OM, and ODE (1:1:2 v/v/v) at 260 ^{o}C.

General procedure for the preparation of ligand-free nanoparticles. The as-prepared oleic acid-capped nanoparticles were extracted from the cyclohexane dispersion and re-dispersed in equal volume of HCl solution (0.1 M in deionized water). The slurry was then sonicated at 45 ^{o}C for 1 h to remove the surface oleate ligands. After the reaction, the nanoparticles were collected via centrifugation at 14000 rpm for 30 min and washed with ethanol and deionized water several times, and re-dispersed in deionized water.

General materials characterization: Transmission electron microscopy (TEM) measurements were carried out on a FEI / Philips Tecnai 12 BioTWIN transmission electron microscope operating at an acceleration voltage of 120 kV. Powder X-ray diffraction (XRD) data were recorded on a Bruker AXS D2 phaser with a graphite-monochro-matized Cu Kα radiation (1.5406 Å). High-resolution TEM and electron energy loss spectroscopy (EELS) mapping analysis were performed on a JEOL-JEM 2100F transmission electron microscope operating at an acceleration voltage of 200 kV. Photoluminescence spectra in the UV and visible range were obtained from water dispersion of the nanoparticles on an F-4600 spectrophotometer (Hitachi), in conjunction with a 980-nm laser diode (continuous wave, 20 W cm^{-2}). Optical gain spectra and lasing spectra were collected under excitation with a 980-nm pulse laser (6 ns, 10Hz) of varying peak powers. Lifetime measurement was performed on UK Edinburgh Instruments FLS900 fluorescence spectrometer with a time-correlated single-photon counting (TCSPC) module, while a 980-nm pulse laser (6 ns, 10 Hz) produced by an OPO was used as the excitation source. The effective lifetimes were determined by

$$\tau_{eff} = \frac{1}{I_0}\int_0^\infty I(t)dt \quad (1)$$

where I_0 and $I(t)$ represents the maximum luminescence intensity and luminescence Intensity at time t after cut off the excitation light, respectively. Photographs of luminescent samples were taken with Nikon D90 camera. Unless otherwise stated, all measurements were carried out at room temperature.

Supplementary Note 2: Monte-Carlo modeling of energy migration

The energy migration process is described using the random-walk model. The Monte-Carlo calculation of the random walk was executed using C++ and performed in a simulated 3D coordinate matrix.

In crystalline materials, each atom can be simplified as a lattice point L and the migration of excitation energy only occurs among the nearest neighboring atoms. For hcp $NaYbF_4$ crystal, an excited ion initially located at a lattice point "*o*" has three types of neighbors, i.e. *a*, *b*, and *c* as shown in the figure below. Due to the short Yb-Yb separations (r_{oa}=3.47 Å, r_{ob}=3.84 Å, r_{oc}=5.93 Å), the Yb-Yb migration is dominated by an exchange energy transfer and the probability of each migration P is proportional to exp(-2*r*/0.3), where *r* is the distance of the movement. Accordingly, we have the corresponding migration probability $P_{o\to a}=11.78\cdot P_{o\to b}>>P_{o\to c}$. So the migration only occurs from site *o* to site *a* or from site *o* to site *b*. In total for each step, there are 8 kinds of displacements (the coordinate is built as shown in the figure below)

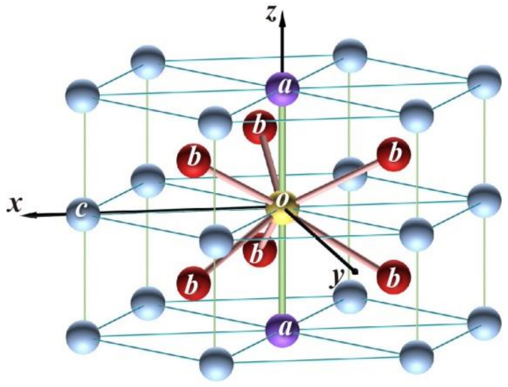

$$\vec{d}_i = \begin{cases} (0,0,\pm r_{oa}) \text{ with the probability } P_{o\to a}\text{, case 1, 2} \\ (0,-\frac{\sqrt{3}}{3} r_{oc},\pm \frac{r_{oa}}{2}) \text{ with the probability } P_{o\to b} \text{ each, case 3, 4} \\ (\pm \frac{r_{oc}}{2}, \frac{\sqrt{3}}{6} r_{oc},\pm \frac{r_{oa}}{2}) \text{ with the probability } P_{o\to b} \text{ each, case 5 - 8} \end{cases}$$

The Monte-Carlo simulation can then be generated using the following criteria,

(1) Every excitation energy initiates at lattice point $L(t{=}0)=(0, 0, \mathrm{m})$, where m is nearest number to $(r_1 + r_2) / 2$ with $\mathrm{m} = k\mathrm{r}_{\mathrm{oa}}$ (k is an positive integer), and r_1 and r_2 are the inner and outer radius of the Yb shell.

(2) The migration proceeds for 5000 steps. For each step of migration, we generate a random number R between 0 and 1 and let $2\cdot P_{\mathrm{o}\rightarrow\mathrm{a}}+6\cdot P_{0\rightarrow\mathrm{b}}=1$,

If $0 < R < P_{\mathrm{o}\rightarrow\mathrm{a}}$, the migration goes to case 1

If $P_{0\rightarrow\mathrm{a}} < R < 2P_{0\rightarrow\mathrm{a}}$, the migration goes to case 2

If $2P_{0\rightarrow\mathrm{a}}+(i\text{-}3)P_{0\rightarrow\mathrm{b}} < R < 2P_{0\rightarrow\mathrm{a}}+iP_{0\rightarrow\mathrm{b}}$, the migration goes to case i, where i=3,4,5,6,7,8.

In case of the boundary situation, some direction(s) may not be available, then probabilities of all remaining directions will be normalized accordingly.

(3) After 5000 steps, the final migration position $L(t{=}5000)$ is recorded.

(4) To generate sufficient samples, in total 10,000,000 independent energy migration undergo random walk.

In Figure 2c, we plot the probability of finding the excitation energy with $|L_{\mathrm{y}}|<6$ to the y=0 plan for three cases r_1=13 nm, r_2=16 nm, 19 nm, and 25 nm. With r_2 increases, the energy migrates to a larger area and the probability of finding the excitation energy in the vicinity of the starting point drops significantly.
